# Efficacy of epidermal growth factor receptor targeting in advanced chordoma: case report and literature review

**DOI:** 10.1186/1471-2407-11-423

**Published:** 2011-10-04

**Authors:** Simon G Launay, Bruno Chetaille, Fanny Medina, Delphine Perrot, Serge Nazarian, Jérôme Guiramand, Laurence Moureau-Zabotto, François Bertucci

**Affiliations:** 1 Department of Medical Oncology, Institut Paoli-Calmettes, 232 bd Ste-Marguerite, Marseille, 13009, France; 2 Department of Pathology, Institut Paoli-Calmettes, 232 bd Ste-Marguerite, Marseille, 13009, France; 3 Department of Radiology, Institut Paoli-Calmettes, 232 bd Ste-Marguerite, Marseille, 13009, France; 4 Department of Surgery, Hopital Conception, 147 bd Baille, Marseille, 13385, France; 5 Department of Surgical Oncology, Institut Paoli-Calmettes, 232 bd Ste-Marguerite, Marseille, 13009, France; 6 Department of Radiotherapy, Institut Paoli-Calmettes, 232 bd Ste-Marguerite, Marseille, 13009, France; 7 University of Mediterranea, 58 bd Charles Livon, Marseille, 13001, France

## Abstract

**Background:**

Chordomas are very rare low-grade malignant bone tumors that arise from the embryonic rests of the notochord. They are characterized by slow growth and long history with frequent local relapses, and sometimes metastases. While chemotherapy is not efficient, imatinib has shown antitumor activity.

**Case presentation:**

We report on a 76-year-old patient with EGFR-overexpressing advanced chordoma that progressed on imatinib and subsequently responded to erlotinib during 12 months.

**Conclusions:**

We report the fourth case of advanced chordoma treated with an EGFR inhibitor. We also review the literature concerning the rationale and potential of EGFR targeting in chordoma.

## Background

Chordomas are very rare malignant bone tumors (approximate incidence rate 0.1/100.000/year), usually arising in the sacrum, skull base and spine. Median age at diagnosis is 60 years [1]. Originating in the embryonic rests of notochord, chordomas show a dual epithelial-mesenchymal differentiation [2]. Classically, they are low-grade malignancies characterized by slow growth and a long history with frequent local relapses. Thus, surgery is the most common treatment, followed by radiation therapy in case of non-complete resection. After a long local evolution, chordomas can also give rise to metastases (20-30%), generally with low growth potential, primarily in the lungs, but also bones and liver. Chemotherapy has been frustratingly inactive in chordoma [1], and until recently, best supportive care was the only therapeutic option in advanced disease. However, the ongoing elucidation of the molecular mechanisms underlying chordomas has led to new therapeutic hopes. Imatinib, which blocks PDGFRs and KIT activation [3], showed antitumor activity alone [4], then in combination with cisplatin chemotherapy [5] or mTOR inhibitor [6]. Erlotinib (Tarceva, Hoffmann-La Roche Ltd., Basel, Switzerland) is a small molecule tyrosine kinase inhibitor targeting EGFR (epidermal growth factor receptor) in lung cancer [7]. Here, we report on a patient with EGFR-overexpressing advanced chordoma that progressed on imatinib and subsequently responded to erlotinib.

## Case presentation

At first diagnosis, in 1999, the patient was a 65-year old man, Caucasian type, without any specific medical personal or familial history. His medical story began in January with chronic and rebel lumbar pain. In April 1999, pelvic magnetic resonance imaging (MRI) showed a sacral tumor. A distal sacral and coccygeal surgical resection was performed. Histological and immunohistochemical (IHC positivity for CK AE1/AE3, EMA, PS100) analyses confirmed the diagnosis of chordoma obtained by pre-operative biopsy. Post-operative radiotherapy was delivered with a total dose of 60 Grays in 30 fractions.

In April 2006, computed tomography (CT) revealed 3 subcutaneous lesions located behind the left scapula, below the right scapula, and next to the temporal bone. Two lesions (near the left scapula and temporal bone) were surgically removed, and corresponded histologically to typical relapses of chordoma. Two months later, a new recurrence was observed with a right supraclavicular tumor of 2 cm, which was treated by radiotherapy (30 grays in 10 fractions).

In July 2007, a CT scan revealed disease progression with appearance of a multilocular tumor under the left scapula, several infra- and supracentimetric lung nodules suggesting metastases, and an increase in size of the right supraclavicular lesion. Once again, the two soft tissue lesions were surgically removed. Their largest pathological diameters were 9 and 5 cm respectively. Histological analysis confirmed the diagnosis of chordoma. Owing to the positive margins of the peri-scapular lesion, adjuvant radiotherapy was delivered (30 grays in 10 fractions), followed in October 2007 by introduction of imatinib (400 mg/day orally). Treatment was well tolerated. In February 2008, a slowly progressive subcutaneous tumor nodule located under the right scapula was surgically excised. Histological analysis again confirmed the diagnosis of chordoma. Imatinib was continued. Subsequent clinical examination and imaging monitored the stability of the disease until February 2009, at which time a CT scan showed progression at various sites: increase in size of the lung nodules, right cervical adenopathy, and two solid lesions located inside the right pectoralis minor muscle and the right paravertebral back muscle. In spite of an increased dose of imatinib (600 mg daily), the disease continued to progress slowly, even though the patient remained asymptomatic with good performance status.

**Figure 1:**
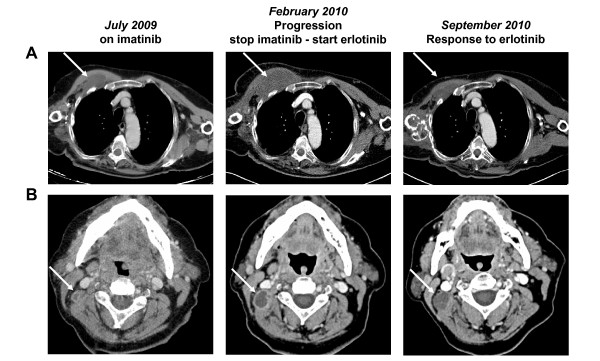
**Cervico-thoracic CT scan showing response to erlotinib**. Three different dates of treatment are shown: July 2009 (on imatinib), February 2010 (progression on imatinib; just before erlotinib), and September 2010: (response after 7 months of erlotinib). Two targets lesions are shown (arrows): the right pectoral lesion (A), and the right cervical adenopathy (B): both progressed between July 2009 and February 2010 during imatinib treatment, and responded to erlotinib between February and September 2010: regression in size of the pectoral lesion (A), and disappearance of the peripheral contrast enhancement for the adenopathy (B).

**Figure 2:**
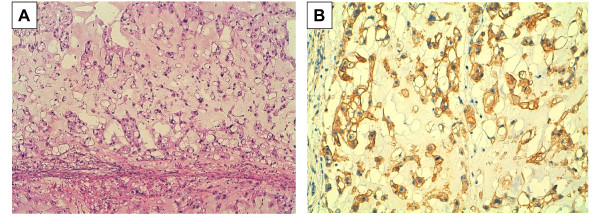
**Chordoma and EGFR overexpression**. A/H&E staining showing round cells with vacuolated cytoplasm arranged in cord-like fashion in a myxoid stroma (original magnification × 100). B/Immunohistochemistry with anti-EGFR antibody showing strong membranous and cytoplasmic staining of tumor cells (original magnification × 200; positivity appears in brown).

Clinical examination and a CT scan in February 2010, revealed further progression, notably regarding the right cervical adenopathy (2.2 × 1.5 cm) and the right pectoral lesion (8.5 × 4.2 cm) (Figure 1). Clinical status deteriorated with a performance status equal to ECOG 1-2, and the appearance of a right anterior thoracic pain. Imatinib was stopped. Recent reports describing antitumor activity of EGFR inhibitors in chordoma [8-10] led us to analyze tumor samples collected from relapses excised in July 2007 and February 2008. Results were similar in both samples. Immunohistochemistry showed a strong EGFR staining in ~90% of tumor cells (Figure 2). Tumor DNA was sequenced and no mutation was discovered in *EGFR, KRAS, BRAF, KIT*, and *PDGFRA*. Fluorescent *in situ *hybridization (FISH) did not indicate *EGFR *amplification, but showed chromosome 7 trisomy in 38% of the tumor cells. Given these results and the absence of any alternative therapeutic option, in February 2010, the 76-year-old patient was started on erlotinib 150 mg daily. After 4 weeks and no side effect, the patient reported a significant improvement of asthenia, and the thoracic pain had completely disappeared. In May 2010, after 3 months of erlotinib, the patient fared very well, the pectoral lesion had decreased, and the CT scan revealed a stabilization of the right cervical adenopathy (2 × 1.5 cm) and a regression by 53% of the pectoral lesion volume (7.6 × 2.3 cm). Lung nodules were stable in size and number. Treatment was continued. In September 2010, a CT scan (Figure 1) showed further regression of the pectoral lesion (6.8 × 1.7 cm; regression of the tumor volume by 70% when compared to February 2010). Other lesions, including the right cervical adenopathy and lung lesions, were stable. Erlotinib was continued without any significant toxicity. In February 2011, after 12 months of erlotinib, all target lesions were clinically and radiologically stable, except the right cervical adenopathy, which progressed slightly (2.1 × 2.8 cm) and was treated by radiotherapy.

## Discussion

There is currently no standard systemic therapy for advanced chordoma, which remains incurable. Recent insights into the molecular biology of the disease have led to new therapeutic hopes, particularly with the demonstration of the antitumor activity of imatinib both *in vitro *and in patients [3-6], and the identification of other potential therapeutic targets such as STAT3 [11], c-MET [12], and brachyury gene [13]. Our present case confirms the relative usefulness of imatinib in advanced chordoma (disease control lasting for nearly two years). More importantly, it points out the potential interest of another tyrosine kinase inhibitor, erlotinib, which resulted in an impressive clinical and radiological response lasting 12 months.

Three cases of advanced chordoma benefiting from EGFR inhibitors have been reported to date in the literature: two were treated with the gefitinib-cetuximab combination [8,9], and one with erlotinib alone [10]. These cases and ours are summarized in Table 1. Despite long delays between initial diagnosis and the anti-EGFR treatment (4 years when used in first line [8,10] and 11 years when used in second (our case) and third lines [9]), clinical benefits and objective tumor regressions were observed in all cases. Responses were long lasting (4+, 9+, 11+, and 12 months), and while they primarily concerned local relapses, they also implicated lung, node, and subcutaneous metastases. In two cases, the simultaneous use of gefitinib and cetuximab obfuscated the individual anitumor contribution of each, whereas the Singhal case and ours clearly demonstrate the activity of erlotinib alone without association with the anti-EGFR antibody. Treatment was well tolerated; it was better for the case of erlotinib alone (no interruption for toxicity) than for the cetuximab-gefitinib combination (interruption lasting 2 and 4 weeks for skin toxicity). Altogether, these observations suggest that a small molecule tyrosine kinase inhibitor could very likely be sufficient.

**Table 1:** Four patients with advanced chordoma treated with EGFR targeting therapy

**Ref**.	Sex	Location of primary tumor	Date of initial diagnosis	Date of beginning of EGFR-targeting therapy	Tumor locations	Patient's age	Therapy	Tumor response	Duration of response
[8]	M	Sacral	2001	2005	Local relapse, Inguinal lymph node, and lung metastases	52	Cetuximab-Gefitinib	« Significant regression » of all lesions after 2 months*	Ongoing after 9 months
[9]	F	Cervical	1996	2007	Local relapse	75	Cetuximab-Gefitinib	Regression by 44% of the volume after 4 months	Ongoing after 4 months
[10]	M	Sacral	2004	2008	Gluteal mass and iliac lymph nodes	57	Erlotinib	Partial response superior to 30% reduction in tumor bulk after 3 months	Ongoing after 11 months
Our case	M	Sacral	1999	2010	Lung metastases, cervical lymph node and thoracic soft tissue mass	76	Erlotinib	Regression of the thoracic lesion by 53% of the volume after 3 months, by 70% after 7 months.	12 months

M, male; F, female; *, the size of lesions and degree of objective response are not mentioned

The rationale for treating chordoma with EGFR inhibitors is inspired by recent reports. Weinberger *et al *showed strong and correlated IHC expression of EGFR and c-MET in a series of 12 chordomas [14]. A Polish study of 21 cases found low to high EGFR expression in 81% of cases and gene amplification in 27% of cases [15]. Activation of the EGFR signaling pathway was reported in a series of 22 clinical samples [16]. The largest series analyzed to date (173 clinical samples) confirmed frequent EGFR expression (69%), and high-level EGFR polysomy in 38% of cases [17]. Phospho-receptor tyrosine kinase analysis showed EGFR activation in the U-CH1 chordoma cell line and all of the three chordomas analyzed. Direct sequencing of *EGFR, KRAS, NRAS, HRAS*, and *BRAF *failed to reveal mutations in 62 cases. The EGFR inhibitor tyrphostin (AG 1478) inhibited proliferation of the U-CH1 cell line *in vitro *and diminished EGFR phosphorylation in a dose-dependant manner, a finding supported by inhibition of phosphorylated Erk1/2 and p-Akt. Altogether, these data clearly implicate aberrant EGFR signaling in the pathogenesis of chordoma. Biological analysis of our case confirmed these findings, with strong EGFR expression, and absence of gene mutation. FISH analysis revealed trisomy of chromosome 7 in 38% of tumor cells, an alteration already reported in chordomas and frequently associated with c-MET overexpression [18]. In our case, whether the trisomy of chromosome 7 is the causal mechanism of EGFR overexpression is not certain.

## Conclusion

Our case highlights the antitumor activity of EGFR inhibition in advanced chordoma, in agreement with three other clinical cases previously reported and pre-clinical data. The targeting of EGFR represents an attractive challenge in chordoma, which clearly calls for prospective assessment in multicentric clinical trials.

## Consent

Written informed consent was obtained from the patient for publication of this case report and any accompanying images. A copy of the written consent is available for review by the Editor-in-Chief of this journal.

## List of abbreviations

CT: computed tomography; EGFR: epidermal growth factor receptor; IHC: immunohistochemistry; MRI: magnetic resonance imaging; PDGFR: platelet-derived growth factor receptor

## Competing interests

The authors declare that they have no competing interests.

## Authors' contributions

Conception and design: FB; Manuscript writing: SGL and FB; Final approval: FB, SGL, BC, FM, SN, DP, JG, LMZ; Pathological explorations: BC; Patient's management: FB, SN, FM, LMZ

## Pre-publication history

The pre-publication history for this paper can be accessed here:

http://www.biomedcentral.com/1471-2407/11/423/prepub
